# (4a*R*,9*R*,9a*R*)-7-Bromo-9-nitro­methyl-2,3,4,4a,9,9a-hexa­hydro-1*H*-xanthen-1-one

**DOI:** 10.1107/S1600536813008659

**Published:** 2013-04-27

**Authors:** Chao Wu, Yan-Jun Guo, Ai-Bao Xia

**Affiliations:** a State Key Laboratory Breeding Base of Green Chemistry-Synthesis Technology, Zhejiang University of Technology, Hangzhou 310014, People’s Republic of China

## Abstract

The title compound, C_14_H_14_BrNO_4_, contains a tricyclic ring system including three contiguous stereocenters all of which exhibit an *R* configuration. The cyclo­hexa­none ring adopts a chair conformation. The central oxane ring assumes a strained envelope conformation, with five of the ring atoms being nearly coplanar with the bromo­phenyl group and with the C atom adjacent to the O atom and fused with the cyclo­hexa­none ring as the flap. In the crystal, mol­ecules are linked into a three-dimensional network by C—H⋯O inter­actions.

## Related literature

For related structures, see: Shi *et al.* (2004 ▶); Xia *et al.* (2009 ▶); Ndjakou Lenta *et al.* (2007 ▶). For background information on domino reactions, see Enders *et al.* (2007 ▶); Yu & Wang (2002 ▶).
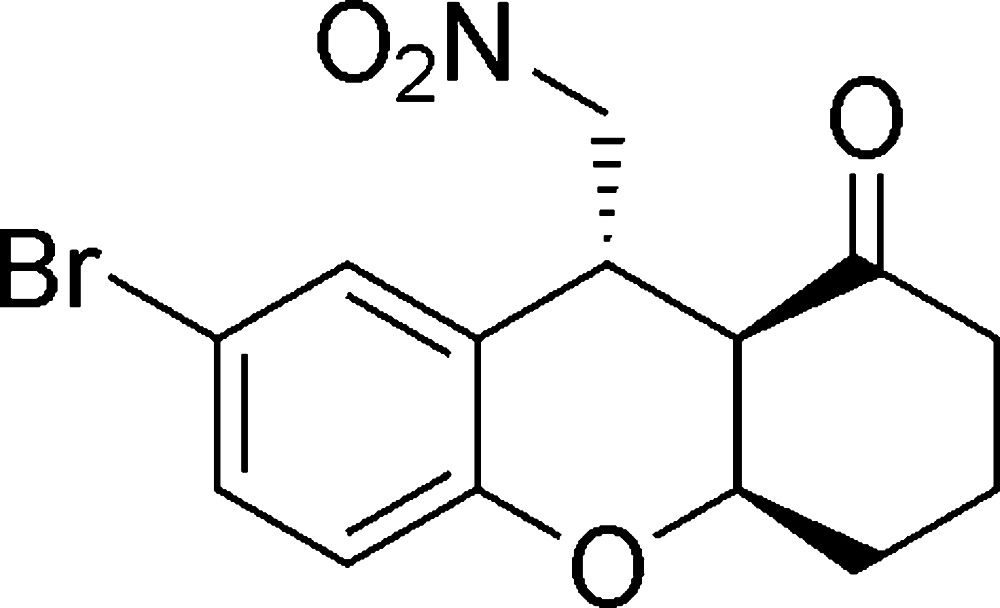


## Experimental

### 

#### Crystal data

C_14_H_14_BrNO_4_
*M* *_r_* = 340.17
Monoclinic, 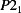

*a* = 10.3457 (7) Å
*b* = 5.4662 (5) Å
*c* = 13.2446 (12) Å
β = 102.849 (2)°
*V* = 730.25 (11) Å^3^
*Z* = 2
Mo *K*α radiation
μ = 2.83 mm^−1^
*T* = 296 K
0.53 × 0.47 × 0.14 mm

#### Data collection

Rigaku R-AXIS RAPID diffractometer
Absorption correction: multi-scan (*ABSCOR*; Higashi, 1995 ▶) *T*_min_ = 0.224, *T*_max_ = 0.673
6335 measured reflections
2655 independent reflections
1537 reflections with *I* > 2σ(*I*)
*R*_int_ = 0.061

#### Refinement

*R*[*F* ^2^ > 2σ(*F* ^2^)] = 0.052
*wR*(*F* ^2^) = 0.137
*S* = 1.00
2655 reflections
181 parameters
1 restraint
H-atom parameters constrained
Δρ_max_ = 0.36 e Å^−3^
Δρ_min_ = −0.46 e Å^−3^
Absolute structure: Flack (1983 ▶), 1058 Friedel pairs
Flack parameter: 0.021 (17)

### 

Data collection: *PROCESS-AUTO* (Rigaku, 2006 ▶); cell refinement: *PROCESS-AUTO*; data reduction: *CrystalStructure* (Rigaku, 2007 ▶); program(s) used to solve structure: *SHELXS97* (Sheldrick, 2008 ▶); program(s) used to refine structure: *SHELXL97* (Sheldrick, 2008 ▶); molecular graphics: *ORTEP-3 for Windows* (Farrugia, 2012 ▶); software used to prepare material for publication: *WinGX* (Farrugia, 2012 ▶).

## Supplementary Material

Crystal structure: contains datablock(s) global, I. DOI: 10.1107/S1600536813008659/fy2090sup1.cif

Structure factors: contains datablock(s) I. DOI: 10.1107/S1600536813008659/fy2090Isup2.hkl

Supplementary material file. DOI: 10.1107/S1600536813008659/fy2090Isup3.cml

Additional supplementary materials:  crystallographic information; 3D view; checkCIF report

## Figures and Tables

**Table 1:** Hydrogen-bond geometry (Å, °)

*D*—H⋯*A*	*D*—H	H⋯*A*	*D*⋯*A*	*D*—H⋯*A*
C3—H3⋯O1^i^	0.93	2.71	3.520 (9)	146
C8—H8*B*⋯O2^ii^	0.97	2.59	3.489 (6)	155
C8—H8*A*⋯O4^iii^	0.97	2.54	3.253 (4)	131
C10—H10⋯O2^iv^	0.98	2.53	3.300 (8)	135
C11—H11⋯O3^v^	0.98	2.59	3.547 (8)	165

Symmetry codes: (i) 
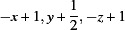
; (ii) 
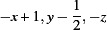
; (iii) 
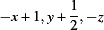
; (iv) 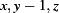; (v) 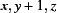.

**Fig. 1.**
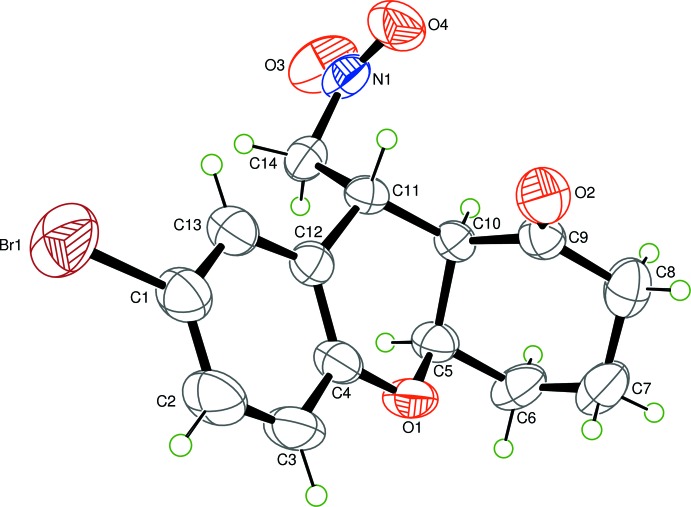
The asymmetric unit of the structure of the title compound, with the atomic labeling scheme. Displacement ellipsoids are drawn at the 50% probability level.

**Fig. 2.**
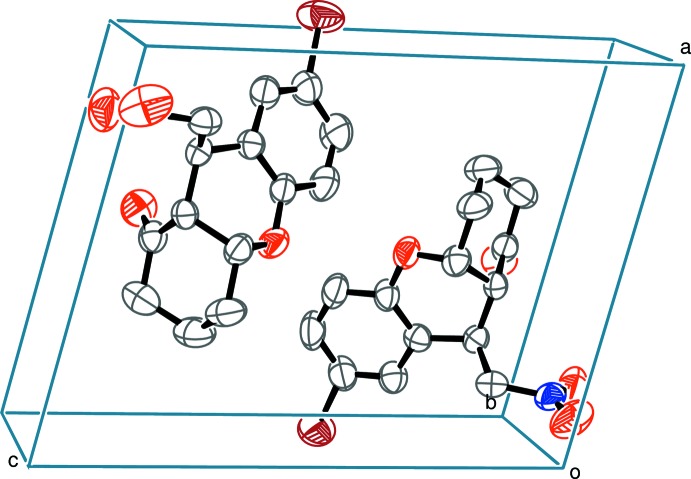
Unit cell packing of the title compound.

## supplementary crystallographic information

### Comment

Domino or cascade reactions, in which multiple new bonds and stereocenters could be formed, have been getting more interest in asymmetric organocatalysis (Enders *et al.*, 2007; Yu *et al.*, 2002) . The title compound, (I), was synthesized as one of a series of oxa-Michael-Michael products under investigation.

In this paper, the crystal of title compound (4aR,9*R*,9aR)-7-bromo-9- (nitromethyl)-2,3,4,4a,9,9a-hexahydro-1*H*-xanthen-1-one was determined. The structure of (I) is shown in Fig. 1. The O1—C4 bond and the bromophenyl group are almost coplanar: the angle is 3.2 (6) ° between the plane of the bromophenyl ring and the O1—C4—C12 plane. The C11—C12 bond and the bromophenyl plane enclose an angle of 9.4 (7) °. The cyclohexanone ring (C5—C6—C7—C8—C9—C10) adopts a chair conformation. The conformation of the oxane ring is nonstandard. Five atoms of the oxane ring are coplanar with the bromophenyl group and only C5 deviates significantly from the common plane.

### Experimental

To the solution of the catalyst (*S*)-2-(pyrrolidin-2-ylmethylthio)pyridine (20 mol%) and 4-(trifluoromethyl)benzoic acid (10 mol%) in saturated aqueous NaCl (0.25 ml) was added sequentially cyclohexenone (0.8 mmol) and (*E*)-4-bromo-2-(2-nitrovinyl)phenol (0.2 mmol) at room temperature with vigorous stirring. After completion, the reaction mixture was extracted with EtOAc (3*10 ml), washed with water, dried and concentrated. The residue was purified by flash chromatography to the product. Then, suitable crystals of the title compound were obtained by slow evaporation of methanol solution at room temperature.

### Refinement

H atoms were placed in calculated position with C—H=0.98 Å(*sp*), C—H=0.97 Å(sp2), C—H=0.93 Å(aromatic). All H atoms were included in the final cycles of refinement as riding mode, with *U*_iso_(H)=1.2*U*_eq_ of the carrier atoms.

### Crystal data

**Table:**

C_14_H_14_BrNO_4_	*F*(000) = 344
*M**_r_* = 340.17	*D*_x_ = 1.547 Mg m^−^^3^
Monoclinic, *P*2_1_	Mo *K*α radiation, λ = 0.71073 Å
Hall symbol: P 2yb	Cell parameters from 4391 reflections
*a* = 10.3457 (7) Å	θ = 3.2–27.4°
*b* = 5.4662 (5) Å	µ = 2.83 mm^−^^1^
*c* = 13.2446 (12) Å	*T* = 296 K
β = 102.849 (2)°	Platelet, colorless
*V* = 730.25 (11) Å^3^	0.53 × 0.47 × 0.14 mm
*Z* = 2	

### Data collection

**Table:**

Rigaku R-AXIS RAPID diffractometer	2655 independent reflections
Radiation source: rotating anode	1537 reflections with *I* > 2σ(*I*)
Graphite monochromator	*R*_int_ = 0.061
Detector resolution: 10.00 pixels mm^-1^	θ_max_ = 26.0°, θ_min_ = 3.2°
ω scans	*h* = −12→12
Absorption correction: multi-scan (*ABSCOR*; Higashi, 1995)	*k* = −6→6
*T*_min_ = 0.224, *T*_max_ = 0.673	*l* = −16→16
6335 measured reflections	

### Refinement

**Table:**

Refinement on *F*^2^	Secondary atom site location: difference Fourier map
Least-squares matrix: full	Hydrogen site location: inferred from neighbouring sites
*R*[*F*^2^ > 2σ(*F*^2^)] = 0.052	H-atom parameters constrained
*wR*(*F*^2^) = 0.137	*w* = 1/[σ^2^(*F*_o_^2^) + (0.0525*P*)^2^] where *P* = (*F*_o_^2^ + 2*F*_c_^2^)/3
*S* = 1.00	(Δ/σ)_max_ < 0.001
2655 reflections	Δρ_max_ = 0.36 e Å^−^^3^
181 parameters	Δρ_min_ = −0.46 e Å^−^^3^
1 restraint	Absolute structure: Flack (1983), 1058 Friedel pairs
Primary atom site location: structure-invariant direct methods	Flack parameter: 0.021 (17)

### Special details

**Table:**

Geometry. All e.s.d.'s (except the e.s.d. in the dihedral angle between two l.s. planes) are estimated using the full covariance matrix. The cell e.s.d.'s are taken into account individually in the estimation of e.s.d.'s in distances, angles and torsion angles; correlations between e.s.d.'s in cell parameters are only used when they are defined by crystal symmetry. An approximate (isotropic) treatment of cell e.s.d.'s is used for estimating e.s.d.'s involving l.s. planes.
Refinement. Refinement of *F*^2^ against ALL reflections. The weighted *R*-factor *wR* and goodness of fit *S* are based on *F*^2^, conventional *R*-factors *R* are based on *F*, with *F* set to zero for negative *F*^2^. The threshold expression of *F*^2^ > σ(*F*^2^) is used only for calculating *R*-factors(gt) *etc*. and is not relevant to the choice of reflections for refinement. *R*-factors based on *F*^2^ are statistically about twice as large as those based on *F*, and *R*- factors based on ALL data will be even larger.

### Fractional atomic coordinates and isotropic or equivalent isotropic displacement parameters (Å^2^)

**Table:**

	*x*	*y*	*z*	*U*_iso_*/*U*_eq_	
C1	0.1121 (7)	0.9599 (12)	0.3568 (5)	0.0740 (18)	
C2	0.2242 (7)	0.9776 (13)	0.4388 (6)	0.084 (2)	
H2	0.2201	1.0658	0.4980	0.101*	
C3	0.3408 (7)	0.8631 (13)	0.4308 (5)	0.079 (2)	
H3	0.4154	0.8757	0.4847	0.095*	
C4	0.3469 (5)	0.7291 (10)	0.3426 (5)	0.0575 (14)	
C5	0.4665 (5)	0.4397 (10)	0.2629 (5)	0.0594 (15)	
H5	0.4212	0.2917	0.2785	0.071*	
C6	0.6112 (5)	0.3829 (12)	0.2656 (6)	0.074 (2)	
H6A	0.6535	0.3222	0.3337	0.089*	
H6B	0.6160	0.2555	0.2156	0.089*	
C7	0.6840 (5)	0.6043 (15)	0.2415 (5)	0.0816 (19)	
H7A	0.7741	0.5588	0.2397	0.098*	
H7B	0.6882	0.7244	0.2960	0.098*	
C8	0.6170 (5)	0.7183 (13)	0.1378 (6)	0.077 (2)	
H8A	0.6599	0.8722	0.1292	0.092*	
H8B	0.6273	0.6101	0.0820	0.092*	
C9	0.4716 (5)	0.7627 (10)	0.1311 (4)	0.0553 (14)	
C10	0.3944 (4)	0.5445 (8)	0.1576 (4)	0.0461 (12)	
H10	0.3945	0.4183	0.1050	0.055*	
C11	0.2504 (4)	0.6049 (10)	0.1573 (4)	0.0487 (12)	
H11	0.2196	0.7266	0.1029	0.058*	
C12	0.2391 (5)	0.7152 (9)	0.2586 (5)	0.0518 (14)	
C13	0.1217 (6)	0.8341 (11)	0.2703 (5)	0.0650 (16)	
H13	0.0473	0.8255	0.2160	0.078*	
C14	0.1623 (5)	0.3767 (10)	0.1314 (5)	0.0572 (15)	
H14A	0.2038	0.2403	0.1732	0.069*	
H14B	0.0773	0.4073	0.1485	0.069*	
N1	0.1410 (4)	0.3111 (10)	0.0188 (5)	0.0621 (14)	
O1	0.4667 (3)	0.6210 (10)	0.3413 (3)	0.0647 (10)	
O2	0.4213 (4)	0.9576 (7)	0.1075 (4)	0.0745 (13)	
O3	0.1104 (4)	0.0988 (9)	−0.0041 (4)	0.1026 (17)	
O4	0.1497 (5)	0.4689 (10)	−0.0439 (4)	0.0874 (14)	
Br1	−0.04348 (7)	1.1196 (2)	0.36550 (7)	0.1208 (5)	

### Atomic displacement parameters (Å^2^)

**Table:**

	*U*^11^	*U*^22^	*U*^33^	*U*^12^	*U*^13^	*U*^23^
C1	0.094 (4)	0.080 (4)	0.048 (5)	0.010 (3)	0.015 (4)	−0.003 (4)
C2	0.096 (5)	0.101 (5)	0.057 (5)	0.017 (4)	0.022 (4)	−0.021 (4)
C3	0.091 (5)	0.100 (5)	0.042 (4)	0.014 (4)	0.003 (3)	−0.012 (4)
C4	0.064 (3)	0.065 (3)	0.044 (4)	0.008 (3)	0.015 (3)	0.007 (3)
C5	0.063 (3)	0.065 (3)	0.050 (4)	0.008 (3)	0.011 (3)	0.009 (3)
C6	0.045 (3)	0.093 (4)	0.078 (5)	0.008 (3)	−0.002 (3)	−0.024 (4)
C7	0.046 (3)	0.098 (5)	0.096 (5)	0.001 (4)	0.004 (3)	−0.019 (5)
C8	0.069 (4)	0.080 (4)	0.087 (6)	0.000 (3)	0.029 (4)	−0.008 (4)
C9	0.066 (3)	0.061 (4)	0.037 (4)	−0.004 (3)	0.008 (3)	−0.005 (3)
C10	0.053 (2)	0.047 (3)	0.039 (3)	0.000 (2)	0.011 (2)	−0.009 (2)
C11	0.057 (2)	0.044 (2)	0.044 (3)	−0.002 (3)	0.009 (2)	0.005 (3)
C12	0.053 (3)	0.054 (3)	0.050 (4)	0.003 (2)	0.013 (2)	0.006 (2)
C13	0.070 (3)	0.066 (4)	0.059 (5)	−0.001 (3)	0.013 (3)	0.010 (3)
C14	0.047 (3)	0.056 (3)	0.069 (5)	−0.006 (2)	0.013 (3)	0.001 (3)
N1	0.037 (2)	0.072 (3)	0.073 (4)	0.011 (2)	0.002 (2)	−0.010 (3)
O1	0.063 (2)	0.086 (2)	0.040 (2)	0.016 (2)	0.0012 (16)	−0.008 (2)
O2	0.084 (3)	0.056 (2)	0.083 (4)	−0.013 (2)	0.017 (2)	0.011 (2)
O3	0.097 (3)	0.070 (3)	0.123 (4)	−0.004 (3)	−0.015 (3)	−0.040 (3)
O4	0.104 (3)	0.105 (4)	0.052 (3)	−0.013 (3)	0.013 (3)	−0.012 (3)
Br1	0.1011 (5)	0.1479 (8)	0.1168 (8)	0.0504 (6)	0.0314 (5)	−0.0231 (7)

### Geometric parameters (Å, º)

**Table:**

C1—C13	1.359 (9)	C7—H7B	0.9700
C1—C2	1.405 (9)	C8—C9	1.506 (8)
C1—Br1	1.857 (6)	C8—H8A	0.9700
C2—C3	1.383 (8)	C8—H8B	0.9700
C2—H2	0.9300	C9—O2	1.196 (6)
C3—C4	1.393 (8)	C9—C10	1.519 (7)
C3—H3	0.9300	C10—C11	1.525 (6)
C4—O1	1.376 (6)	C10—H10	0.9800
C4—C12	1.391 (7)	C11—C12	1.499 (7)
C5—O1	1.435 (8)	C11—C14	1.538 (7)
C5—C6	1.521 (7)	C11—H11	0.9800
C5—C10	1.538 (8)	C12—C13	1.417 (7)
C5—H5	0.9800	C13—H13	0.9300
C6—C7	1.497 (10)	C14—N1	1.502 (8)
C6—H6A	0.9700	C14—H14A	0.9700
C6—H6B	0.9700	C14—H14B	0.9700
C7—C8	1.527 (9)	N1—O4	1.214 (6)
C7—H7A	0.9700	N1—O3	1.223 (7)
			
C13—C1—C2	119.0 (6)	C7—C8—H8B	109.3
C13—C1—Br1	121.1 (5)	H8A—C8—H8B	108.0
C2—C1—Br1	119.8 (5)	O2—C9—C8	122.0 (5)
C3—C2—C1	119.6 (6)	O2—C9—C10	122.6 (5)
C3—C2—H2	120.2	C8—C9—C10	115.4 (5)
C1—C2—H2	120.2	C9—C10—C11	113.1 (4)
C2—C3—C4	120.3 (6)	C9—C10—C5	109.1 (4)
C2—C3—H3	119.8	C11—C10—C5	111.1 (4)
C4—C3—H3	119.8	C9—C10—H10	107.8
O1—C4—C3	116.4 (5)	C11—C10—H10	107.8
O1—C4—C12	122.0 (5)	C5—C10—H10	107.8
C3—C4—C12	121.5 (5)	C12—C11—C10	110.8 (4)
O1—C5—C6	106.3 (5)	C12—C11—C14	111.4 (4)
O1—C5—C10	108.8 (4)	C10—C11—C14	110.8 (4)
C6—C5—C10	111.9 (5)	C12—C11—H11	107.9
O1—C5—H5	109.9	C10—C11—H11	107.9
C6—C5—H5	109.9	C14—C11—H11	107.9
C10—C5—H5	109.9	C4—C12—C13	116.2 (5)
C7—C6—C5	111.7 (5)	C4—C12—C11	122.0 (5)
C7—C6—H6A	109.3	C13—C12—C11	121.5 (5)
C5—C6—H6A	109.3	C1—C13—C12	123.3 (6)
C7—C6—H6B	109.3	C1—C13—H13	118.3
C5—C6—H6B	109.3	C12—C13—H13	118.3
H6A—C6—H6B	107.9	N1—C14—C11	111.3 (5)
C6—C7—C8	111.8 (5)	N1—C14—H14A	109.4
C6—C7—H7A	109.2	C11—C14—H14A	109.4
C8—C7—H7A	109.2	N1—C14—H14B	109.4
C6—C7—H7B	109.2	C11—C14—H14B	109.4
C8—C7—H7B	109.2	H14A—C14—H14B	108.0
H7A—C7—H7B	107.9	O4—N1—O3	124.0 (6)
C9—C8—C7	111.5 (6)	O4—N1—C14	119.5 (5)
C9—C8—H8A	109.3	O3—N1—C14	116.4 (6)
C7—C8—H8A	109.3	C4—O1—C5	116.7 (4)
C9—C8—H8B	109.3		
			
C13—C1—C2—C3	−1.3 (11)	C5—C10—C11—C14	−84.5 (5)
Br1—C1—C2—C3	−178.5 (6)	O1—C4—C12—C13	179.8 (5)
C1—C2—C3—C4	−0.6 (11)	C3—C4—C12—C13	−3.2 (9)
C2—C3—C4—O1	−179.9 (6)	O1—C4—C12—C11	−6.6 (8)
C2—C3—C4—C12	2.9 (11)	C3—C4—C12—C11	170.4 (6)
O1—C5—C6—C7	−61.4 (7)	C10—C11—C12—C4	−6.8 (7)
C10—C5—C6—C7	57.2 (7)	C14—C11—C12—C4	117.0 (5)
C5—C6—C7—C8	−55.4 (7)	C10—C11—C12—C13	166.4 (4)
C6—C7—C8—C9	51.6 (7)	C14—C11—C12—C13	−69.8 (6)
C7—C8—C9—O2	128.3 (7)	C2—C1—C13—C12	1.0 (10)
C7—C8—C9—C10	−51.1 (7)	Br1—C1—C13—C12	178.1 (4)
O2—C9—C10—C11	−3.6 (8)	C4—C12—C13—C1	1.2 (9)
C8—C9—C10—C11	175.8 (5)	C11—C12—C13—C1	−172.4 (6)
O2—C9—C10—C5	−127.8 (6)	C12—C11—C14—N1	162.8 (4)
C8—C9—C10—C5	51.6 (6)	C10—C11—C14—N1	−73.3 (5)
O1—C5—C10—C9	63.7 (5)	C11—C14—N1—O4	−25.2 (6)
C6—C5—C10—C9	−53.5 (6)	C11—C14—N1—O3	157.5 (4)
O1—C5—C10—C11	−61.7 (5)	C3—C4—O1—C5	166.4 (6)
C6—C5—C10—C11	−178.9 (5)	C12—C4—O1—C5	−16.5 (8)
C9—C10—C11—C12	−83.4 (6)	C6—C5—O1—C4	170.2 (5)
C5—C10—C11—C12	39.7 (6)	C10—C5—O1—C4	49.5 (7)
C9—C10—C11—C14	152.4 (5)		

### Hydrogen-bond geometry (Å, º)

**Table:**

*D*—H···*A*	*D*—H	H···*A*	*D*···*A*	*D*—H···*A*
C3—H3···O1^i^	0.93	2.71	3.520 (9)	146
C8—H8*B*···O2^ii^	0.97	2.59	3.489 (6)	155
C8—H8*A*···O4^iii^	0.97	2.54	3.253 (4)	131
C10—H10···O2^iv^	0.98	2.53	3.300 (8)	135
C11—H11···O3^v^	0.98	2.59	3.547 (8)	165

Symmetry codes: (i) −*x*+1, *y*+1/2, −*z*+1; (ii) −*x*+1, *y*−1/2, −*z*; (iii) −*x*+1, *y*+1/2, −*z*; (iv) *x*, *y*−1, *z*; (v) *x*, *y*+1, *z*.
